# Supplementary material for: A good tennis player does not lose matches. The effects of valence congruency in processing stance-argument pairs
Source: PLoS One. 2019 Nov 5;14(11):e0224481. doi: 10.1371/journal.pone.0224481 (PMC6830817; doi:10.1371/journal.pone.0224481)
Supplement: S1 Appendix — (DOCX) [file pone.0224481.s001.docx]

**S1 Appendix: Experimental materials Study 1**

Table S1 displays a translated version of our experimental materials; the original Dutch materials are available on demand. In these original materials, the length of each stance-argument pairs ranged between 10 and 14 words, of which 4 to 5 words were used for the stance and 6 to 9 for the argument. This way, the stance could be displayed on the first line and the argument on the second line on the screen. All stance-argument pairs conveyed the performance and evaluation of an animate (*N* = 8) or inanimate (*N* = 7) object. For 8 items the argument included some sort of percentage, while in the other 7 items absolute numbers were used.

There are various criteria available to determine what is the marked and what is the unmarked part in a pair of opposites, e.g., the positive pair is often linguistically unmarked, it is used for asking neutral questions, it is the pair part that is named first when the pair of opposites is mentioned and it is often evaluatively positive (see [S1: 1] for an elaborate discussion). We used the latter criterion to label some terms as positive and others as negative as people can make a good distinction in evaluative polarity (see [S1: 2]) and valence scores are available for a large number of Dutch and English words (see [S1: 3], [S1: 4]).

**Table S1. Experimental materials used in Study 1.**

| Statement | Variations | | | |
| --- | --- | --- | --- | --- |
|  | 1 | 2 | 3 |  |
| He is a (1) tennis player  He has (3) (2) of the 25 matches | Good - Bad | 5 – 20 | Won - Lost |  |
| She is a (1) woman  She has answered (2) of the questions (3) | Smart - Dump | 90% - 10% | Correctly - Incorrectly |  |
| He is a (1) man  (2) of the women think he is (3) | Handsome - Ugly | 80% - 20% | Attractive - Unattractive |  |
| She is a (1) doctor  (2) out of 10 patients is (3) | Reliable - Unreliable | 8 – 2 | Satisfied - Dissatisfied |  |
| He is a (1) architect  (2) of the people think the design is (3) | Good - Bad | 85% - 15% | Beautiful - Ugly |  |
| She is a (1) shooter  (2) of the 15 shots are (3) | Good - Bad | 12 – 3 | Hit - Missed |  |
| He is a (1) lawyer  He has (3) (2) of the cases | Smart - Dump | 70% - 30% | Won - Lost |  |
| She is a (1) baker  (2) of the 20 guests think the pie is (3) | Good - Bad | 18 - 2 | Tasteful - Tasteless |  |
| The washing product works (1)  (2) out of 5 users would (3) it | Well - Bad | 1 -4 | Recommend – Advise against |  |
| The book is (1)  (2) of the readers think it is (3) | Interesting - Boring | 65% - 35% | Exciting - Boring |  |
| The exam is (1)  (2) of the pupils has (3) | Easy - Difficult | 90% - 10% | Passed - Failed |  |
| The hotel is (1)  (2) of the 50 guests are (3) | Clean - Dirty | 45 – 5 | Satisfied – Dissatisfied |  |
| The festival is (1)  (2) of the visitors is (3) | Nice - Stupid | 80% - 20% | Having a good time - bored |  |
| The vacuum cleaner works (1)  (2) of the 15 users are (3) | Good Bad | 13 – 2 | Satisfied – Dissatisfied |  |
| The restaurant is (1)  (2) of the guests think the food is (3) | Good - Bad | 70% - 30% | Tasteful - Tasteless |  |

References in this Appendix:

[S1: 1] Horn LR. A natural history of negation. Chicago: Chicago University Press; 1989.

[S1: 2] Moors A, De Houwer J, Hermans D, Wanmaker S, Van Schie K, Van Harmelen A, De Schryver M, De Winne J, Brysbaert M. Norms of valence, arousal, dominance, and age of acquisition for 4300 Dutch words. Behavior Research Methods. 2013; 45: 169-77.

[S1: 3] Warriner AB, Kuperman V, Brysbaert M. Norms of valence, arousal, and dominance for 13,915 English lemmas. Behavior research methods*.* 2013; 45: 1191-1207.

[S1: 4] Saerle SR. Linear models for unbalanced data. New York: Wiley; 2006.
