# Supplementary material for: A good tennis player does not lose matches. The effects of valence congruency in processing stance-argument pairs
Source: PLoS One. 2019 Nov 5;14(11):e0224481. doi: 10.1371/journal.pone.0224481 (PMC6830817; doi:10.1371/journal.pone.0224481)
Supplement: S2 Appendix — (DOCX) [file pone.0224481.s002.docx]

**S2 Appendix: Formalization of the multi-level models used**

Equation S2 displays the model used to estimate the accuracy scores. In the model, *Y_(jk)_* indicates whether individual *j* (*j* = 1, 2, …99) has made a correct judgment about the truth value of item *i* (*i* = 1, 2,….6). Four mean accuracy scores are estimated (β_1_- β_4_), one for every combination of the valence of the stance (Positive or Negative) and the valence of the argument (Positive or Negative). This means that we have used a so-called “cell means model”, so a model that estimates a separate mean for each experimental condition [S2: 1]. The estimated accuracy scores are allowed to vary between persons (*u*_1j_ –*u*_4j)_ and items (*v*_0_*_k_*). This person and item variance are estimated simultaneously, which means that a cross-classified model is in operation (see [S2: 2] [S2: 3]). All residuals are normally distributed with a variance of S^2^*_u_*_1_*_j_* - S^2^*_u_*_4_*_j_*_,_ and S^2^*_v_*_0_*_k_* respectively.

Note that in Logit models the variance because of the interaction between respondent and item is implied, but not estimated. This is because the interaction variance is directly related to the estimates of the fixed parameters. The interaction variance for Logit Models can be calculated using the formula *p* * *(1 – p*). In this equation *p* represents the estimated proportion of accurate answers in a certain condition.

**Equation S2.**

Logit *Y_(jk)_* =
POSITIVESTANCE_POSITIVEARGUMENT_(_*_jk)_* (β_1 +_ *u*_1j_) +
POSITIVESTANCE_NEGATIVEARGUMENT _(_*_jk)_* (β_2 +_ *u*_2j_) +
NEGATIVESTANCE_POSITIVEARGUMENT_(_*_jk)_* (β_3 +_ *u*_3j_) +
NEGATIVESTANCE_NEGATIVEARGUMENT _(_*_jk)_* (β_4 +_ *u*_4j_) +
*v*_0_*_k_*

The model used for analysing the reaction times is comparable to the above model. The only exception is that the DV in that model is an interval variable and therefore the variance due to the interaction between respondent and item is also estimated.

To calculate the effect size for both the accuracy scores and the reaction times, Cohen’s *d* was used. Cohen’s *d* is a measure for the size of an effect relative to the standard deviation [S2: 4]. Our multi-level models include various variance components and these can all be used separately or in combination with one another to evaluate the size of an effect. We report the effect size relative to the combined person and item variance. We used an online effect size calculator for within-subjects designs hosted by Newfoundland and labrador’s university (https://memory.psych.mun.ca/models/stats/effect_size.shtml) to calculate Cohen’s *d*. In the calculator, we entered the means to be compared and calculated the standard deviation by first adding up the relevant variances (i.e., the respondent and item variance) and then taking the square root. As our models are examples of cell means models, we took the square root of the average variance of the two relevant cell means for the main effects (e.g., √ ((3.88 + 1.46 + 0.04 + 0.04)/2) = 1.64 and √ ((1.15 + 1.55 + 0.04 + 0.04)/2) = 1.18 for the main effect of stance in Study 1). Our effects are never refutable, even relative to this strict measure for the effect size. A Cohen’s *d* between .2 and .5 is classified as small, an effect size between .5 and .8 is medium, and a Cohen’s *d* larger than .8 can be called large [S2: 4].

For both the accuracy scores and the reaction times, we have also used some alternative specifications of the models described here. For both dependent variables, models were also run including the raw (untransformed) data (Model A1), including only respondents (Model A2) or Items (Model A3) within 2 standard deviations from the mean. These models gave very similar results. For the reaction times, models were also run including only times related to accurate answers (Model A4). These models also displayed comparable results. Hence, our findings are robust against different ways of analyzing data.

References in this Appendix

[S2: 1] Saerle SR. Linear models for unbalanced data. New York: Wiley; 2006.

[S2: 2] Quené, H, Van den Bergh H. On multilevel modeling of data from repeated measures designs: A tutorial. Speech Communication. 2004; 43: 103-121.

[S2: 3] Quené, H, Van den Bergh H. Examples of mixed-effects modeling with crossed random effects and with binomial data. Journal of Memory and Language*.* 2008; 59: 413-442.

[S2: 4] Cohen J. Statistical power analysis for the behavioral sciences (second ed.). New Jersey: Lawrence Erlbaum Associates; 1988.
