# Supplementary material for: A good tennis player does not lose matches. The effects of valence congruency in processing stance-argument pairs
Source: PLoS One. 2019 Nov 5;14(11):e0224481. doi: 10.1371/journal.pone.0224481 (PMC6830817; doi:10.1371/journal.pone.0224481)
Supplement: S3 Appendix — (DOCX) [file pone.0224481.s003.docx]

**S3 Appendix: Experimental materials Study 2**

Table S3 provides an English translation of the experimental materials. The original Dutch materials are available on demand. In the original Dutch materials, the stances consisted of 4-5 words, and the arguments consisted of 6-9 words. All items concerned the evaluation of an object or event.

**Table S3. Experimental materials used in Study 2**

| Statement | Variation in wording | | |
| --- | --- | --- | --- |
|  | **1** | **2** | **3** |
| The festival is (1)  (2) of the visitors are (3) | Fun - Dull | 80% - 20% | Amused - Bored |
| The test is (1)  (2) of the people received a (3) score | Easy – Difficult | 75%- 25% | High - Low |
| The match is (1)  (2) of the 50 spectators think the level of the play is (3) | Interesting - Boring | 45 – 5 | High - Low |
| The course is (1)  (2) of the 10 students would (3) it | Easy - Difficult | 8 – 2 | Recommend – Advise against |
| The nose spray is (1) effective  (2) of the complaints is (3) reduced | Largely - Hardly | 90% - 10% | Strongly - Hardly |
| The meeting was (1)  (2) of those present were (3) afterwards | Positive - Negative | 12 – 3 | Happy – Sad |

There are minor differences in the analyses reported on here compared to the ones in [S3: 1]: one set of test items has been excluded here, as we have now realized that this item had been formulated ambiguously. Moreover, the analyses in [S3: 1] were based on 99 participants, which was an error: two of these were test runs of the software. The exclusion of these data did not alter the main outcomes of the study.

Reference in this Appendix

[S3: 1] Mos MBJ, Kamoen N. Een goede tennisser verliest geen wedstrijden. Twee reactietijdenstudies naar het effect van attribuutframing in standpunt-argumentparen. [A good tennis player does not lose matches. Two reaction time studies into the effect of attribute framing in stance-argument pairs]. Tijdschrift voor Communicatiewetenschap 2017; 45: 184-200.
