# Supplementary material for: A good tennis player does not lose matches. The effects of valence congruency in processing stance-argument pairs
Source: PLoS One. 2019 Nov 5;14(11):e0224481. doi: 10.1371/journal.pone.0224481 (PMC6830817; doi:10.1371/journal.pone.0224481)
Supplement: S4 Appendix — (DOCX) [file pone.0224481.s004.docx]

**S4 Appendix: Experimental materials Study 3**

Table S4 below provides an English translation of the experimental materials. We compared the Dutch positive and negative terms in the stance and argument for their length (number of characters), lemma frequency, and exact word frequency. There were no differences between the positive and negatives alternatives for any of those measures (in all cases: *p* > .06).

**Table S4. Experimental materials used in Study 3**

| Statement | Variations | | | |
| --- | --- | --- | --- | --- |
|  | 1 | 2 | 3 |  |
| This tennis player played several tournaments the past period. He has (1) (2) out of 10 matches. He is (3). | Won – Lost | 8 - 2 | Good – Bad |  |
| This student took courses in linguistics and visual communication. He has (1) (2) of his exams. He is (3). | Passed – failed | 90% - 10% | Smart – dumb |  |
| This striker has come from soccer club “The Hits” last year. He (1) (2) out of 10 penalties. The striker is good. | Scored - Missed | 10 -2 | Good – Bad |  |
| The government has been installed last year. They (1) (2) of the asylum requests. The government is (3). | Accepted – rejected | 95% - 5% | Flexible – strict |  |
| The doctor treats both gastric ulcers and kidney stones. She was evaluated (1) by (2) of her patients. The doctor is (3). | Positively - negatively | 95% - 5% | Competent – incompetent |  |
| The wedding cake consists of six layers. It was evaluated to be (1) by (2). The cake was (3). | Delicious - disgusting | 97% - 3% | Tastefull – tasteless |  |
| This year, the festivities took place in Eindhoven. They were considered to be (1) by (2) out of 100 guests. The festival has (3). | Interesting - uninteresting | 98 - 2 | Succeeded – failed |  |
| The gymnast performed an exercise on the floor. She completed (2) out of 10 exercises (1). She is (3). | Decently - Sloppily | 8 - 2 | Good – bad |  |
